# A Multi‐Omic Analysis of Molecular Risk and Resilience Factors in Late‐Onset Alzheimer’s Disease in APOEe4 Carriers

**DOI:** 10.1002/alz.092941

**Published:** 2025-01-03

**Authors:** Yaroslav Markov, Ahana Priyanka, Albert Higgins‐Chen

**Affiliations:** ^1^ Yale Graduate School of Arts & Sciences, New Haven, CT USA; ^2^ Yale University, New Haven, CT USA; ^3^ Yale School of Medicine, New Haven, CT USA

## Abstract

**Background:**

While the apolipoprotein E (APOE) ε4 allele is a well‐known risk factor for late‐onset Alzheimer’s disease (LOAD), not all carriers develop the condition, suggesting the presence of resilience and/or risk factors. The molecular signatures of resilience/risk in the brain, however, have not been thoroughly described, partly due to the scarcity of healthy APOEe4 carriers. This study addresses this gap using a novel multi‐tissue, multi‐omic dataset from the Religious Order Study and Memory and Aging Project cohorts highly enriched in APOEe4 carriers with and without LOAD.

**Method:**

Our dataset encompasses post‐mortem samples from three brain regions – superior temporal gyrus (ST), prefrontal cortex (PFC), and cerebellum (CBM) – which exhibit varied susceptibility to AD pathology. Utilizing Illumina EPIC arrays for DNA methylation profiling, deep whole exome sequencing, and label‐free mass spectrometry for protein quantification, we analyze samples from 23 APOE 2/4, 197 APOE 3/4, 17 APOE 4/4, and 96 APOE 3/3 carriers (age range 65 to 108, median 89). This diverse cohort includes 78 individuals without cognitive symptoms, 81 with mild cognitive impairment, and 174 with clinically diagnosed AD dementia, further stratified by NIA Reagan scores, allowing us to rigorously define AD diagnosis based on both clinical and post‐mortem assessments.

**Result:**

We delineate molecular changes associated with resilience/risk to AD specifically in APOEe4 carriers within each brain region. For example, we find sets of 76, 43, and 74 mutated genes strongly associated with AD resilience/risk in ST, PFC, and CBM respectively, featuring an enrichment in genes important for the extracellular matrix. Mutations in these genes, alongside with alterations in DNA methylation and protein levels, may potentially reflect an array of influences, including genetic, environmental, and aging effects, and could provide crucial insights into intervention strategies. For the next phase of this project we will integrate these molecular signatures identified at individual ‐omic levels using a multimodal machine learning approach to further investigate their relevance and interplay in the context of AD resilience/risk.

**Conclusion:**

This comprehensive study illuminates the molecular landscape of Alzheimer’s disease resilience and risk factors in APOEε4 carriers, setting the stage for future integrative, multi‐omic analyses.

